# The effects of long COVID-19, its severity, and the need for immediate attention: Analysis of clinical trials and Twitter data

**DOI:** 10.3389/fdata.2022.1051386

**Published:** 2022-12-15

**Authors:** Arinjita Bhattacharyya, Anand Seth, Shesh Rai

**Affiliations:** ^1^Department of Bioinformatics and Biostatistics, University of Louisville, Louisville, KY, United States; ^2^SK Patent Associates, LLC, Dublin, OH, United States; ^3^Cancer Biostatistics and Bioinformatics Shared Resource (Cancer BBSR) in the Department of Environmental and Public Health Sciences, University of Cincinnati College of Medicine, Cincinnati, OH, United States

**Keywords:** PASC, COVID-19, coronavirus, clinical trials, Twitter, negative binomial regression, long-COVID-19

## Abstract

**Background:**

The coronavirus disease 2019 (COVID-19) has been declared a pandemic since March 2020 by the World Health Organization; identifying the disease progression, predicting patient outcomes early, the possibility of long-term adverse events through effective modeling, and the use of real-world data are of immense importance to effective treatment, resource allocation, and prevention of severe adverse events of grade 4 or 5.

**Methods:**

First, we raise awareness about the different clinical trials on long COVID-19. The trials were selected with the search term “long COVID-19” available in ClinicalTrials.gov. Second, we curated the recent tweets on long-haul COVID-19 and gave an overview of the sentiments of the people. The tweets obtained with the query term #long COVID-19 consisted of 8,436 tweets between 28 August 2022 and 06 September 2022. We utilized the National Research Council (NRC) Emotion Lexicon method for sentiment analysis. Finally, we analyze the retweet and favorite counts are associated with the sentiments of the tweeters *via* a negative binomial regression model.

**Results:**

Our results find that there are two types of clinical trials being conducted: observational and interventional. The retweet counts and favorite counts are associated with the sentiments and emotions, such as disgust, joy, sadness, surprise, trust, negative, and positive.

**Conclusion:**

We need resources and further research in the area of long COVID-19.

## Background

The coronavirus disease 2019 (COVID-19) was declared a pandemic in March 2020 by the World Health Organization (WHO). There have been 426,551,362 cases and 5,898,442 deaths worldwide since its advent in December 2019 (COVID-19 Map, 2022). The SARS-CoV-2 virus has been transmitted to humans and has produced several deadly and highly infectious variants through mutations. The infection pathway follows symptoms of fever, cough, shortness of breath, and dyspnea, and in some severe cases leading to hospitalization, emergency life support, and even death. Identifying the disease progression and predicting patient outcomes in early stages, precisely predicting the possibility of long-term adverse events by effective modeling, and by using of real-world data, such as clinical trial data, electronic health records data, and health insurance data. These data sets are of immense importance to establish an effective treatment, resource allocation, and prevention of severe adverse events (SAE) in grade 4 or 5. Significant research is needed for such situations, thus helping to allocate the “right drug to the right patient.” The primary goals of this study are to address the current initiatives: (a) through clinical trials of the COVID-19 infection, (b) the prevalence of long-haul COVID-19, and (c) to increase awareness for predicting the immediate and long-term outcome of COVID-19 by using novel prediction algorithms. In addition, there is also a need to assess the importance of demographics, genetic biomarkers, comorbidities, concomitant medication, and social, genetic, environmental, or economic effects in affecting the outcome of the disease.

To support this future research, we intend to use survival, binary logistic, and count regression models depending on the outcome of interest. Furthermore, depending on the availability of data, innovative research can and will be done to develop novel methodologies relating to variable selection and variable selection, such as shrinkage prior methods of Lasso, Elastic-Net, and Bayesian shrinkage estimation. The purpose of applying these methods is to find the most significant variables responsible for the long COVID-19 effect. Finally, whether the person will develop long-term COVID-19 could be predicted. As a part of the research, the groups of patients with similar outcomes will need to be stratified by treatment interventions. The novel techniques would need validation by simulations and applied to real-world electronic health record (EHR) and clinical trial data.

We anticipate age, sex, gender, race, vaccination status, concomitant medications, prior illness, comorbidities, and symptoms to be responsible factors and would contribute toward understanding the disease progression. The short-term outcomes may not only depend on these factors, but additional covariates, such as recovery, time-varied symptoms, genetic factors, interaction effects, hospitalization, and ventilation status, would also be contributing factors.

Based on the accuracy of the methods, the long-term presence of SARS-CoV-2 RNA in humans and its dependency on demographics, genetics, and interaction effects would need to be addressed. The potential long-term consequences of COVID-19 survivors will impact those who are affected and would need public health, government, and health policymakers' attention to mitigate concerns and possibly encourage the development of therapeutics. This research work lays the foundation for future guidance based on the currently available clinical trials. The goal of this research is to understand public opinion toward COVID-19 by analyzing tweets from people across the globe on Twitter and also getting information about the interventional and observational clinical trials from the ClinicalTrials.gov.

Furthermore, conducting subgroup findings through a novel methodology will help to stratify patients having similar results that will help us identify treatment allocation strategies.

Patients with similar outcomes need to be stratified, and treatment interventions need to be allocated accordingly. The novel techniques need validation by simulations and are applied to real-world EHR and clinical trial data.

Based on the accuracy of the methods, the long-term presence of SARS-CoV-2 RNA in humans and its dependency on demographics, genetics, and interaction effects need to be addressed, and the potential long-term consequences for COVID-19 survivors will impact patients and need public health, government, and health policymakers' further attention to mitigate such concerns and will encourage the development of therapeutics. This research is the stepping stone for future guidance and reports the currently available clinical trials and describes them. It also lays the ground for understanding public opinion toward COVID-19 by analyzing tweets from people on the social media.

## Motivation

The COVID-19 pandemic is the most significant global crisis since World War II that affected almost all the countries on earth, according to the United Nations (Feinerer et al., 2008).

As of 27 May 2022, there are 7,695 studies listed concentrating on COVID-19 listed on ClinicalTrials.gov concentrating on COVID-19, of which only 19 are dedicated to understanding the long-term effects of COVID-19. Of the 19 studies, one had the results available, three were completed, one study was enrolling patients by invitation, three were not yet recruiting, one study had been withdrawn, and 10 were currently recruiting. Only one concluded study had posted the results (NCT04871815, Effects of Sodium Pyruvate Nasal Spray in COVID-19 Long Haulers). Eight studies of them were drug studies, and one was device-related. The outcome measures included serious adverse event (SAE), short-term and long-term feature changes the disease, and separation between symptoms of severe vs. non-severe cases, among other outcomes. All the studies included all men and women except one study, which only enrolled the female population (NCT05225220, “multimodal investigation of post-COVID-19 in females”) with a device as an intervention. The sponsors included both universities and hospitals. The median age group was 18 years or older (65 years or more). One of the studies [NCT04956445, “Collection of SARS-CoV-2 (COVID-19) Virus Secretions and Serum for Countermeasure Development”] included children of ages 6 months or older. Most of the studies were either interventional (63%) or observational (31%) out of the total 18 studies. Six studies reached the phase 2 stage of the clinical trial. Of note, 31% of the studies are industry-sponsored. The maximum enrollment was 2,000 patients, and a minimum of 20 enrollees had the least number of participants. Seven study designs are allocated randomly. The earliest start date of a trial was as early as 17 March 2020, with the recent study being on 3 May 2022. Brain fog is one such severe condition, and the neurological effect of exposure to this virus is of grave concern. Only one study focuses on this post-trauma and neurological aspect of COVID-19 (NCT5042466). This necessitates attention toward analytical research where biostatisticians can contribute a lot toward clarifying different aspects of long COVID-19. This research work utilizes ClinicalTrials.gov data and Twitter data to understand the condition of long COVID-19 research and the people's opinions, respectively. We further wanted to conduct this work so that clinicians and researchers are aware of the existing research on long COVID-19 and also the knowabouts of the clinical trials concerning long COVID-19. The trials are summarized, and the findings are uniquely described. Similarly, with the help of the Twitter data, using well-known methods, such as sentiment analysis, we were able to analyze the people's opinions about long COVID-19.

### Existing research

Since the pandemic, there have been many articles; 452 publications existed on COVID-19 from November 2019 to May 2020 (Raynaud et al., 2021), most of which are not focused on long-term COVID infections. Studies have shown that long-haul COVID-19 is prevalent in 25% (1 in 4) of patients with COVID-19, irrespective of severity. Patients with COVID-19, after recovery, continue to suffer for weeks and months. Research can help answer the questions on the long-term consequences of the disease from our patients and healthcare providers. A recent meta-analysis study based on 29 studies on the global prevalence of post-acute sequelae of COVID-19 (PASC) or long COVID showed that there was a global long COVID-19 prevalence estimate of 0.43 (95% confidence interval [CI]: 0.35, 0.63), with women having a high rate of prevalence than men, with regional prevalence having highest in the Asian population followed by Europe and the United States. Among commonly reported PASC symptoms, fatigue and dyspnea were reported most frequently, with a prevalence of 0.23 (95% CI: 0.13, 0.38) and 0.13 (95% CI: 0.09, 0.19), respectively. The global pooled PASC prevalence decreased for 30–90 days after the index test positive date (Chen et al., 2021).

Although it is anticipated that most of the COVID-19 symptoms disappear within 21 days, nearly 10% of patients with COVID-19 show signs even after 3 weeks, 5% for 8 weeks or more, and 2% suffer for almost 3 months. The long-haul patients, after recovering from severe symptoms of shortness of breath, chest pain, most commonly brain fog, fever, and headache, among others, irrespective of the variant of infection, could have permanent damage to the lungs due to acute respiratory distress syndrome (ARDS) and possibly Alzheimer's disease due to the most common-brain fog, pulmonary, cardiovascular, neurological effects, and idiopathic inflammation. To understand this newly developed disease, further research, including the epidemiology, spread, pulmonary biology, neurological, and cardiovascular effects, attention to the need of the affected patients through long-term follow-ups, and the creation of post-COVID clinics are essential.

Some notable research has indicated the impact of nutrition and high-fat diets that lead to diabetes and obesity, contributing to the long-term effects of COVID-19 (Lopez-Leon et al., 2021). More than 50 long-term symptoms were reported in a systematic review, of which the most common are fatigue, headache, attention disorder, hair loss, and dyspnea (Ludvigsson, 2021). Subsequently, more than 100 symptoms have been documented.

Case reports have also suggested that children may experience similar long COVID symptoms, women being more affected (Yelin et al., 2020). One of the significant concerns of long-haul COVID-19 is that the patient is unaware of the long-term effects, which cannot be predicted at the early stage of the disease. The participation of an international and interdisciplinary group of researchers with the availability of big data, such as EHR data sets, clinical trial data, and medical insurance data, provides rich sources to extract the signals. Such data with a combination of novel methodologies will help understand the consequences and characteristics of both local and global COVID-19-affected populations. This global perspective from research studies will be helpful in formulating international health policy and opening COVID-19 long-term clinics and health insurance policies (Butler and Barrientos, 2020; del Rio et al., 2020). Machine learning (ML) and standard statistical models are available for the task, but we need to understand the data in-depth and then address healthcare and health policy needs. However, inference derived from such data-driven methodologies should be cautiously interpreted. Often these models' condition on patients testing positive for COVID-19, but that may introduce bias depending on who has access to testing/care. Choosing the right control group is key to obtaining a valid prediction model. Each prediction model has its pros and cons, and so assumptions must be carefully justified (Mukherjee, 2022).

### Limitations

There are several limitations in the existing research. We are unable to know the global demographics of long COVID-19. Furthermore, it is interesting to see how the patients with long-term COVID-19 are treated and the resources allocated for these patients, whether any genetic, gene-treatment, or gene–environment factors work as effect modifiers responsible for long COVID, and what are the treatment options available for these patients. Existing studies in children and adolescents have considerable limitations, and distinguishing long-term SARS-CoV-2 infection-associated symptoms from pandemic-related symptoms is difficult (Zimmermann et al., 2022).

## Methodology

**Figure 1:**
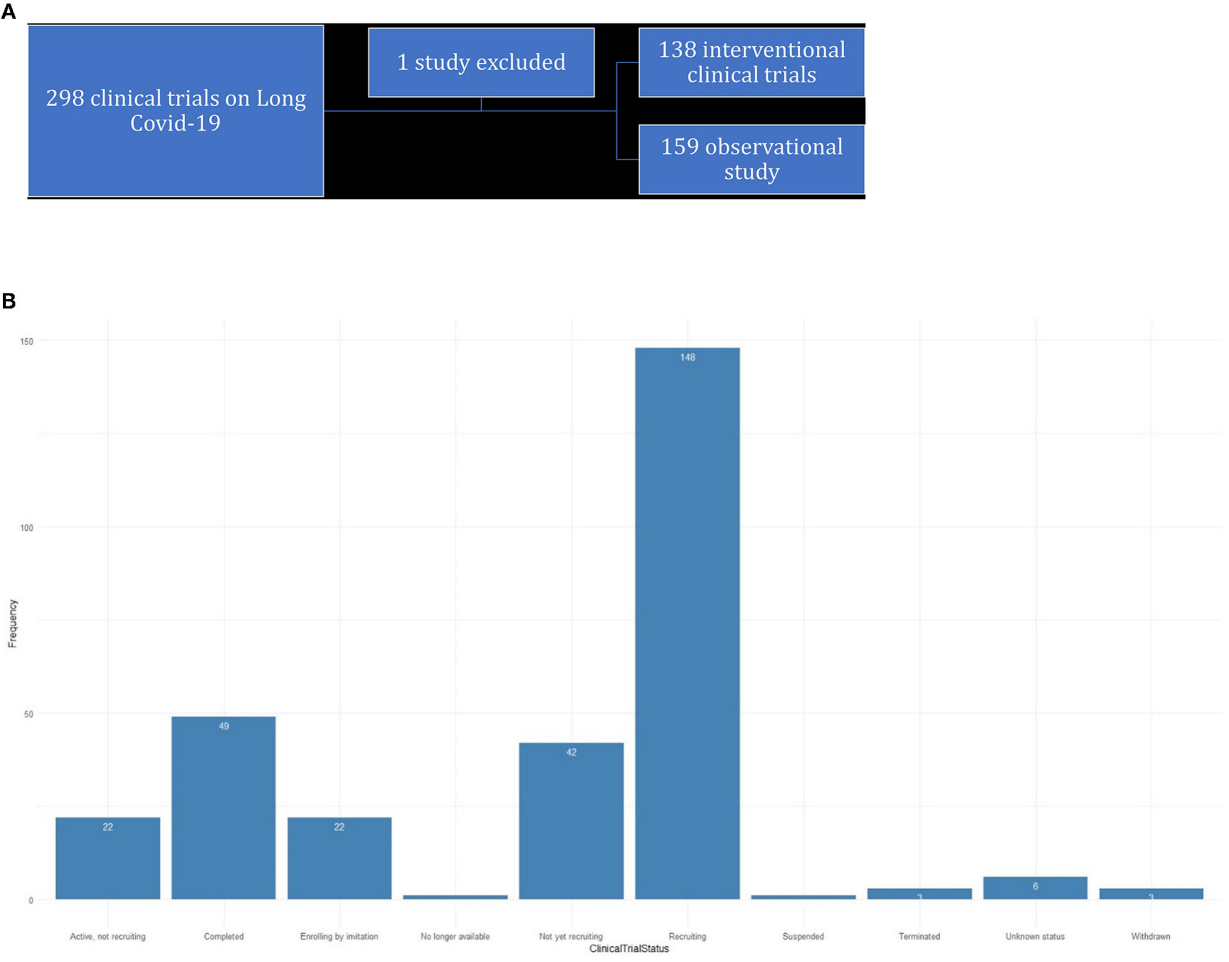
**(A)** Trials curated from ClinicalTrials.gov. **(B)** Status of clinical trails for long-COVID-19.

The main goal of this study is 3-fold: First, we raise awareness about the different clinical trials that are being conducted concurrently on long COVID-19 and how these trials prove beneficial in our understanding of long COVID-19. The trials are curated from ClinicalTrials.gov and chosen with the search term “long COVID-19” (Figure 1A). We mostly analyzed these data using effective data visualization techniques.

Second, we analyzed the recent tweets on long-haul COVID-19 curated from Twitter. The tweets were obtained with the query term #long COVID-19 consisting of 8,436 tweets between 28 August 2022 and 06 September 2022 and gave an overview of the sentiments of the people's opinions. The data were analyzed with the help of RStudio. The R packages were used to address the analysis of the “twitter,” “syuzhet,” and “tm” (Gentry et al., 2016; Feinerer and Hornik, 2022; Title Natural Language Processing Infrastructure, 2022).

One way to analyze the sentiment of a text is to consider the text as a combination of its individual words and the sentiment content of the whole text as the sum of the sentiment content of the individual words. We utilized the “NRC Emotion Lexicon” (EmoLex) method for sentiment analysis. EmoLex is a list of English words and their associations with eight basic emotions (anger, fear, anticipation, trust, surprise, sadness, joy, and disgust) and two sentiments (negative and positive) (NRC Emotion Lexicon, 2022). We obtained a matrix of sentiment scores, retweet counts, and favorite counts that formed the basis of our analysis. We regressed the retweet counts and the favorite counts with the sentiment scores and investigated whether they are associated with the emotions and sentiments of the tweeters *via* a negative binomial regression model since the outcome variable is count data.

### Reporting results on long COVID-19 from ClinicalTrials.gov

As of 5 September 2022, there were 297 studies posted on ClinicalTrials.gov, where research is concentrated toward post-acute sequelae SARS-CoV-2 infection (PASC) or COVID-19. The status of the studies is shown in the bar diagram in Figure 1B.

**Figure 2:**
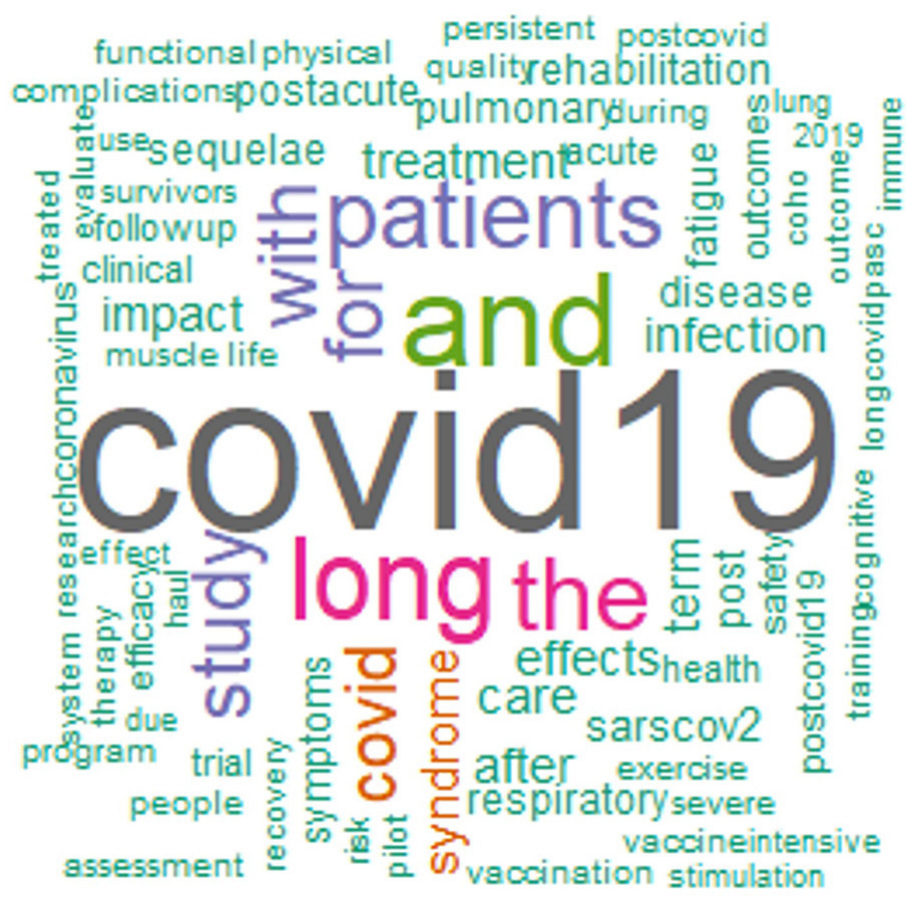
Wordcloud of titles of all the long-COVID-19 trails.

**Figure 3:**
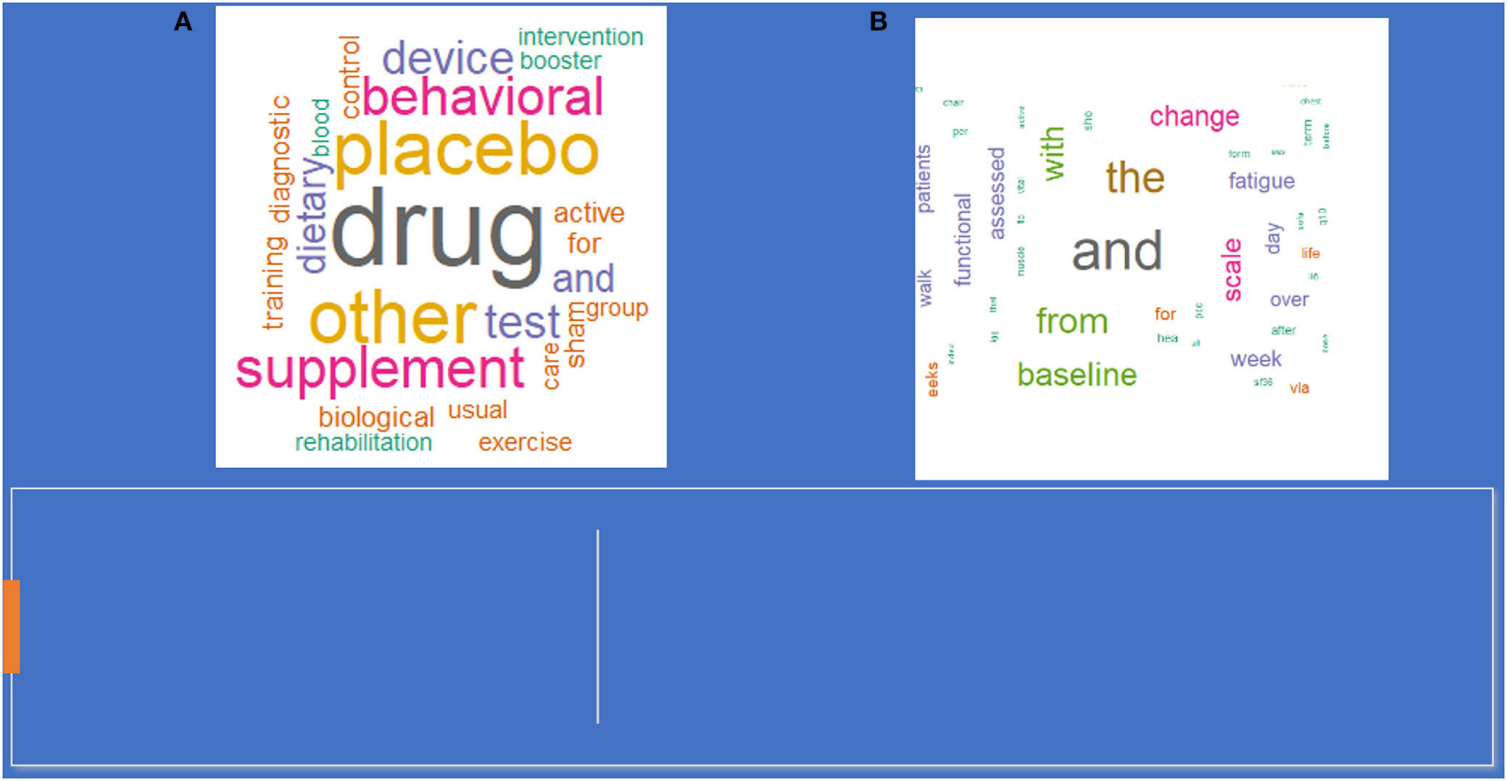
Wordcloud. **(A)** Intervention in the trails. **(B)** Outcome measures of interventional trails.

In 294 trials, both men and women were enrolled, two trials were restricted to male participants, and one study had only for female participants. Figure 2 represents the word cloud of all the long COVID-19 trials. Figure 3 shows the word clouds for (a) titles of interventional trials, (b) use of interventions in the trials, and (c) outcome measures of interventional trials.

There are 159 trials that were categorized as observational or other types. We have excluded the trial “NCT04798066” while analyzing the observational studies as it is neither observational or an interventional trial.

**Figure 4:**
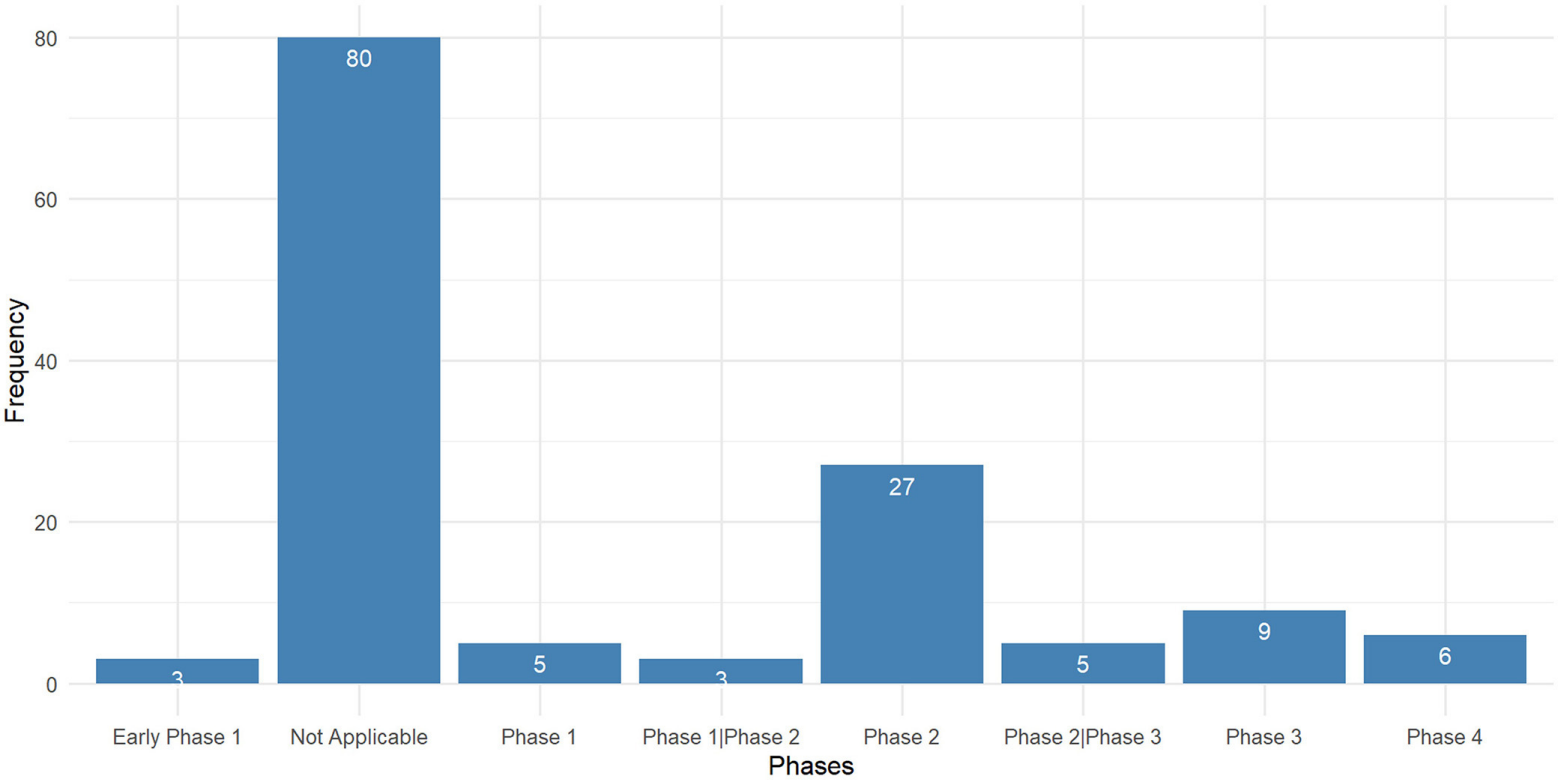
Study type of interventional clinical trails for long-COVID-19.

There were 138 interventional trials, and their clinical trial phases are listed in Figure 4.

### Interventional trials

In the interventional trials, ~48% were 18 years older.

**Figure 5:**
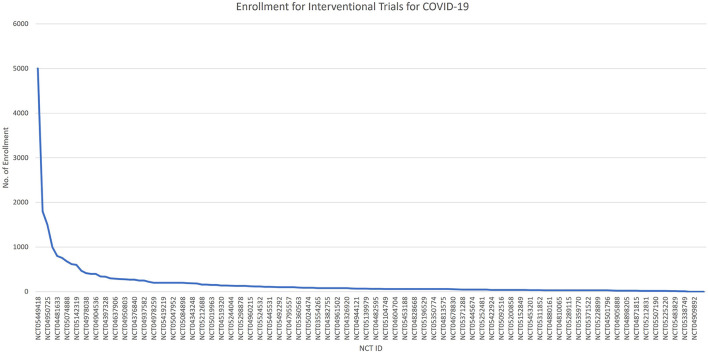
The no. of participants enrolled in interventional trails.

**Figure 6:**
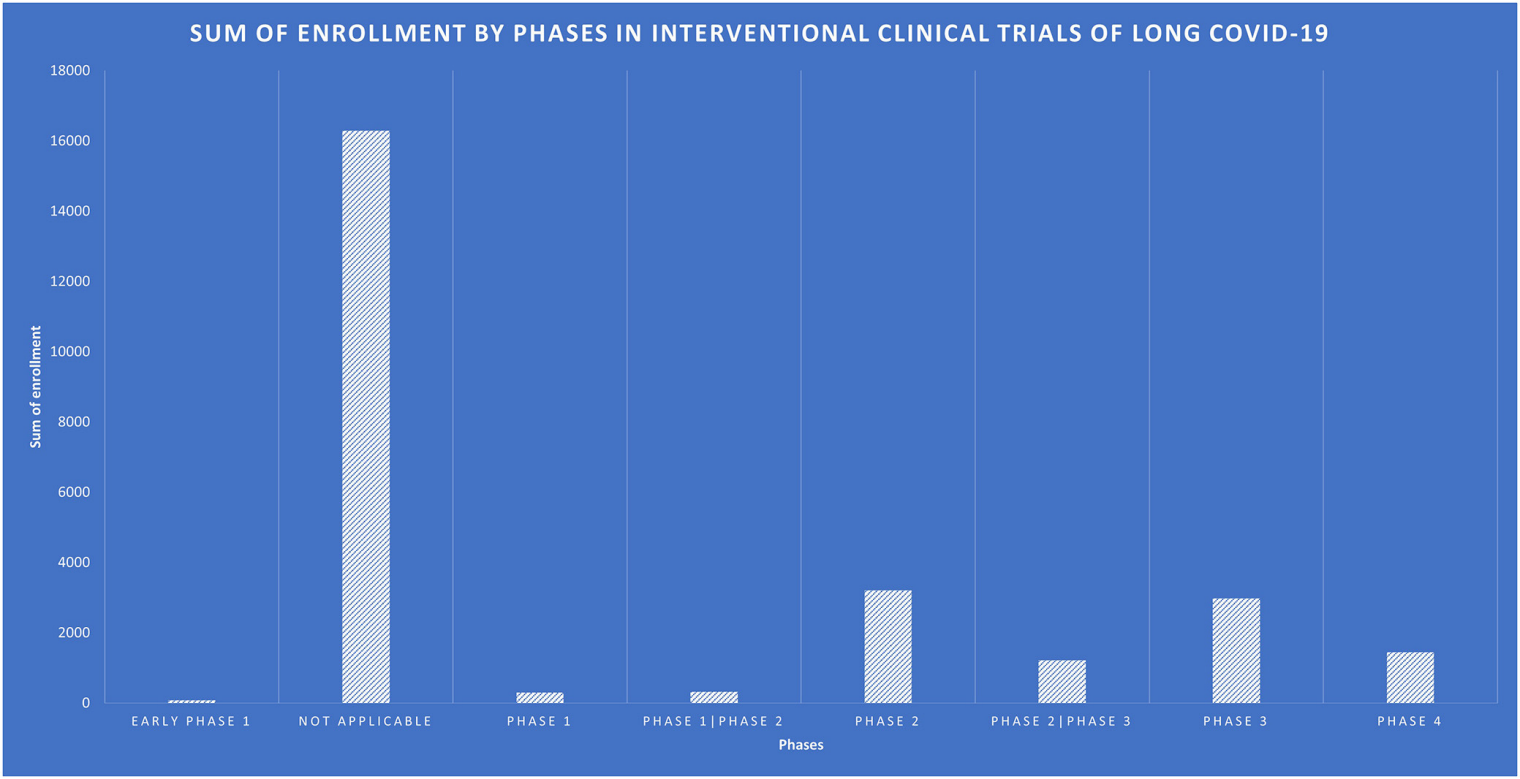
Sum of enrollment by phases in interventional clinical trials of long COVID-19.

The rest of them have some restrictions on the upper limit age. Figure 5 shows the number of participants enrolled in interventional trials by NCT IDs, with NCT05449418 enrolling the largest number of 5,000 participants, followed by NCT05168800, NCT04950725, NCT05513560, and NCT04481633. Figure 6 shows the total number of participants enrolled in the interventional trials by phases.

**Figure 7:**
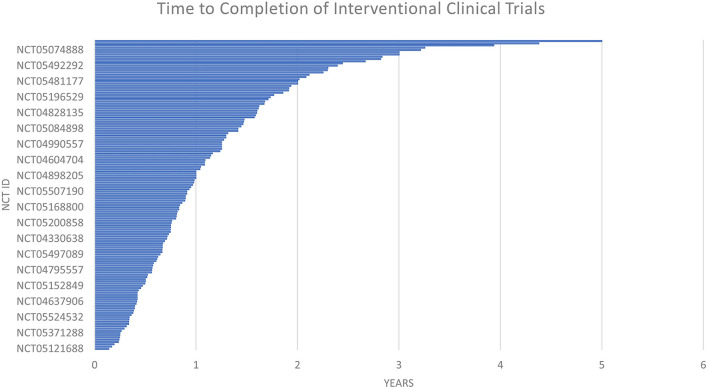
The time to completion of interventional clinical trails.

**Figure 8:**
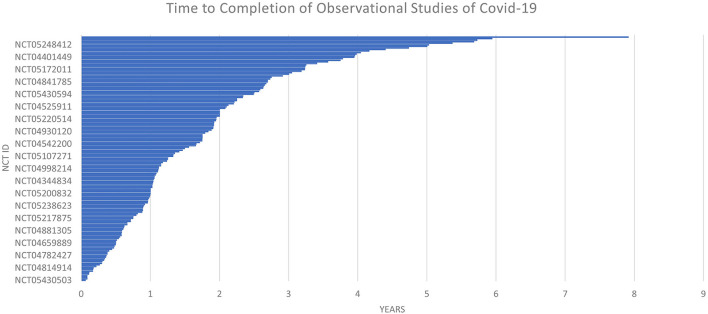
Time to completion of observational studies of COVID-19.

**Figure 9:**
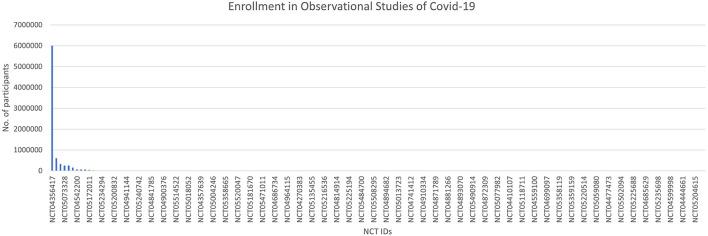
Enrollment in observational studies of COVID-19.

The time to completion of both types of trials (observational and interventional) is given in Figures 7 and 8. The maximum time to completion was 5 years for interventional trials Figure 8. The enrollment in observational studies is present in Figure 9.

### Observational studies

Out of 158 observational studies, one study was restricted to the enrollment of men only. The time for completion for an observational trial was listed as a maximum of ~8 years for an observational trial. The enrollment in one of the observational trials was quite large NCT ID NCT04356417 with a number of participants of 6,000,000.

#### Pediatric trials

There are four interventional trials that include children, and the trial numbers are NCT05373043 (funded by US Federal Government), NCT05445531, NCT04900961, and NCT05084898. The average enrollment is 207.75 participants with no restrictions on age. There are blind as well as open-label trials, with mostly parallel designs. There are 30 observational studies that will include children.

## Analysis of tweets from social media

**Figure 10:**
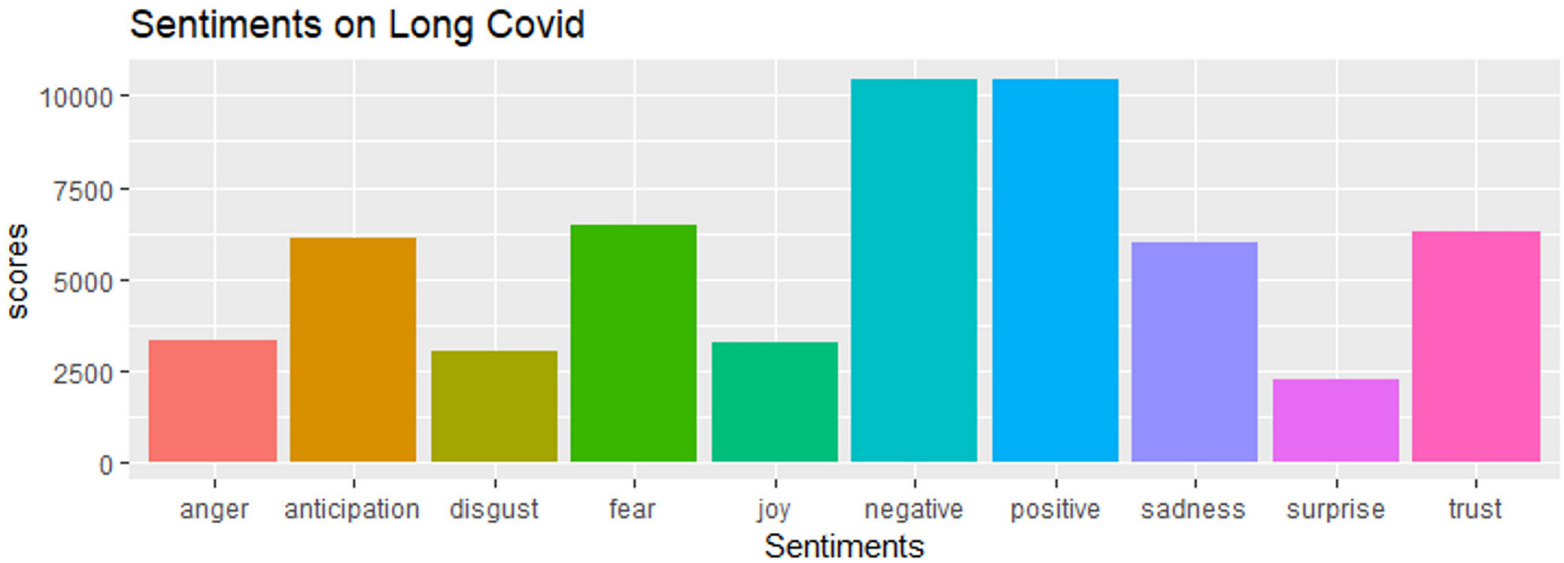
Sentiment analysis of tweets.

**Figure 11:**
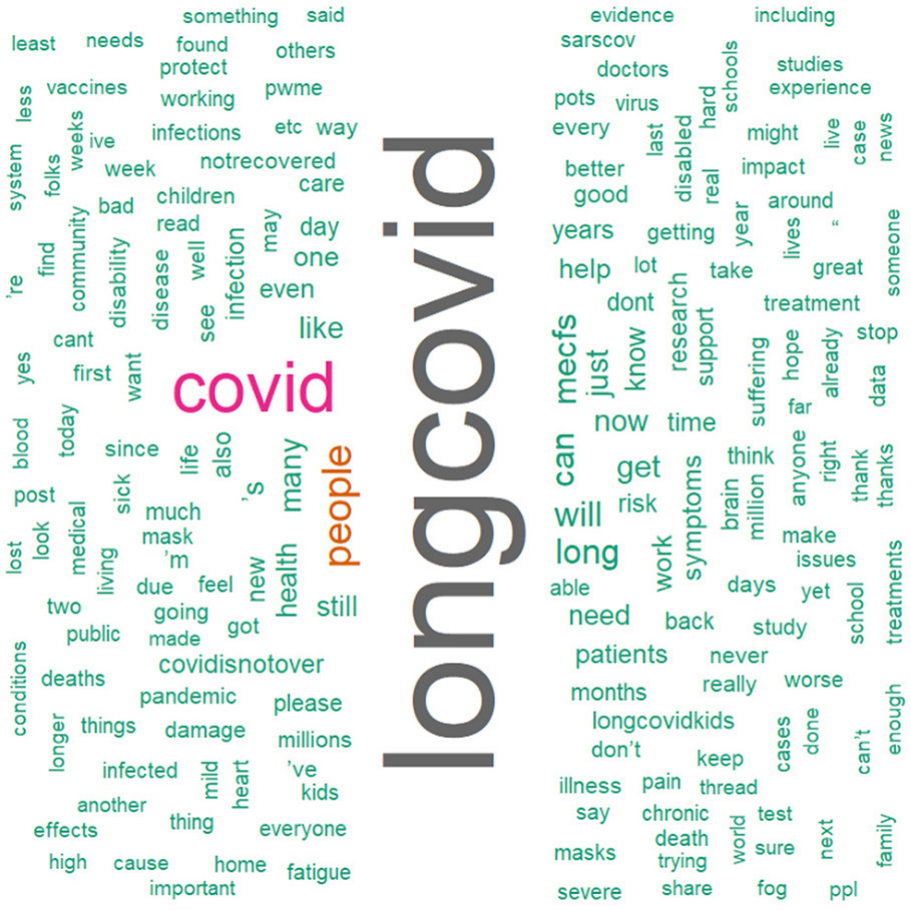
Word Cloud of Twitter data with #long-COVID-19.

**Figure 12:**
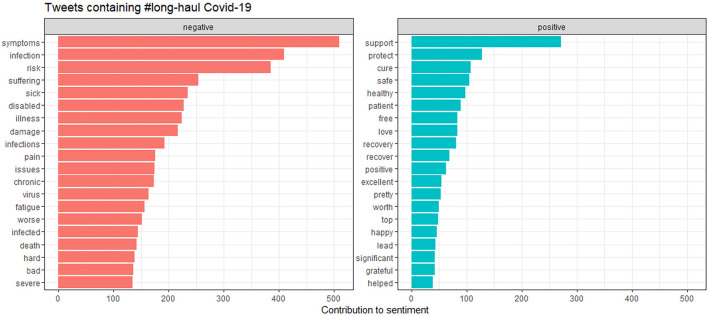
The contribution of positive and negative sentiments.

We conducted a sensitivity analysis with 8,436 tweets generated from the social media platform Twitter and wanted to see the current status of long COVID-19 and people's opinions about it on social media. As we can see that there is a significant amount of both positive and negative sentiments in Figure 10. From Figure 11, the word “long COVID” has the highest frequency, followed by “COVID.” The word cloud gives a better picture in Figure 12.

**Figure 13:**
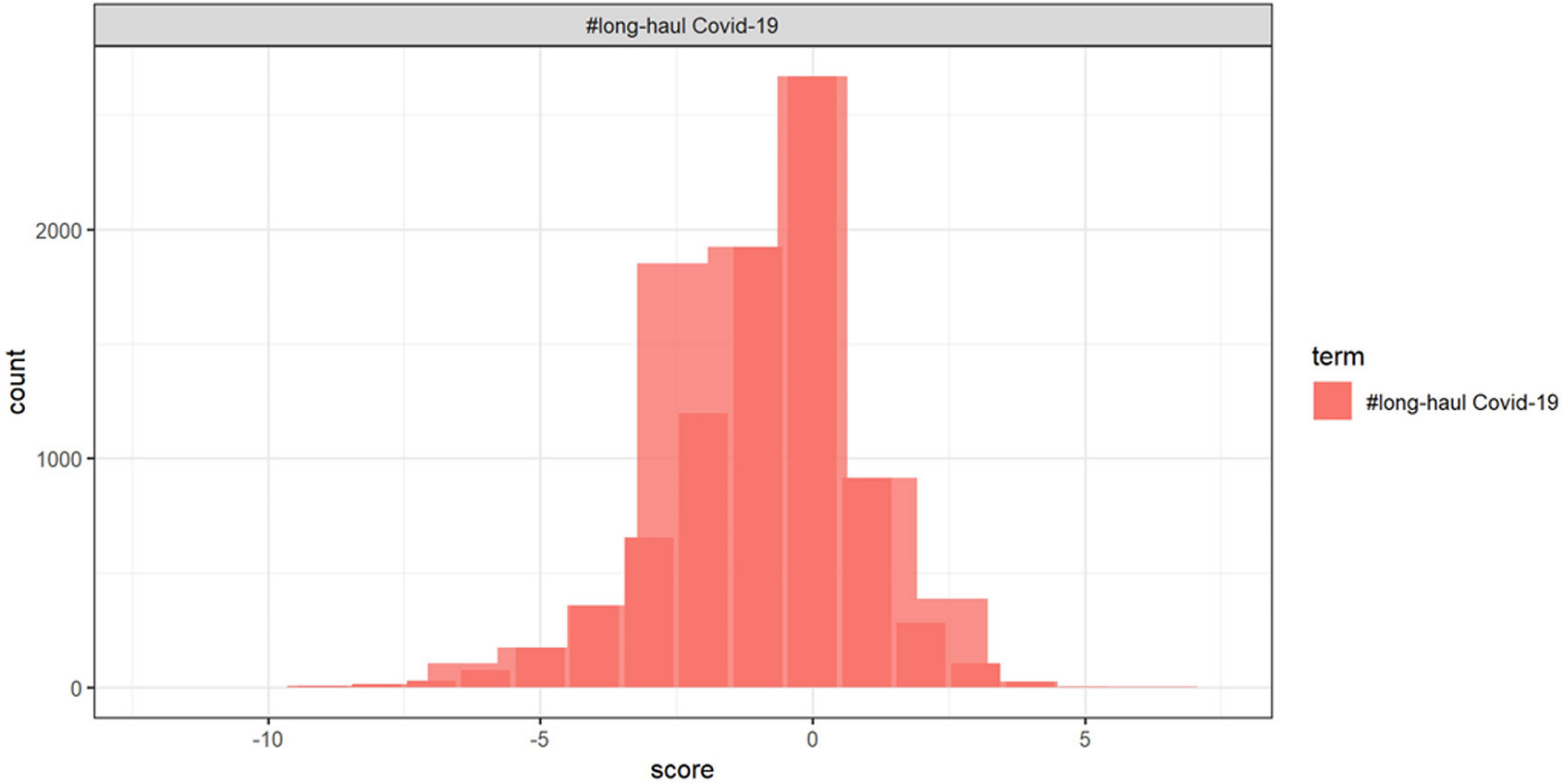
The distribution of sentiment scores.

Figure 12 shows the contribution of words toward positive and negative symptoms, with Figure 13 showing the distribution of scores.

## Analysis of tweet sentiments by negative binomial regression model

After obtaining the sentiment scores (SS) for each of the observations (tweets) through the “NRC” method of sentiment analysis, we tested the following hypotheses:

Hypothesis 1: The retweet counts are dependent on the sentiments.

Hypothesis 2: The favorite (like) counts are dependent on the sentiments.

We achieved these through Poisson, negative binomial, and zero-inflated models. Count data regression modeling has received much attention in several science fields in which the Poisson, negative binomial, and zero-inflated models are some of the primary regression techniques. Negative binomial regression is applied to modeling count variables, usually when they are over-dispersed. A Poisson distribution is used when the mean is equal to the variance. This situation is often unrealistic. The distribution of counts will usually have a variance that is not equal to its mean. Modeling it as Poisson distribution leads to ignoring under- or over-dispersion, depending on if the variance is smaller or larger than the mean. Also, situations with outcomes having a larger number of zeros require special attention, usually handled using zero-inflated models.

**Figure 14:**
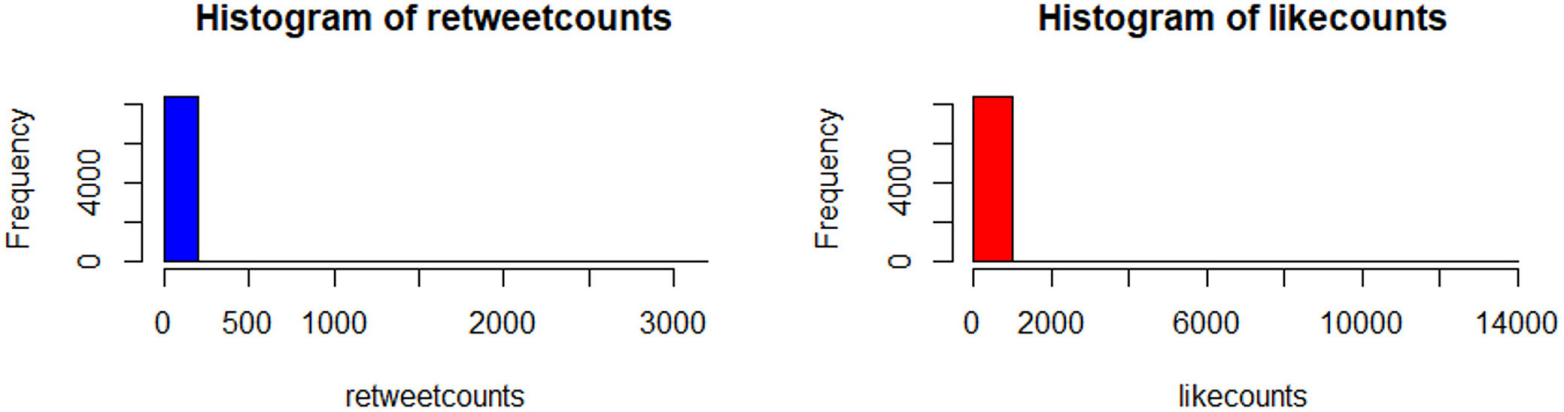
The histogram of the tweets shows an abundance of zeros.

The histogram of the retweet counts and favorite counts is in Figure 14. The histogram shows a high proportion of zeros in the outcome measures. The covariates are “anger,” “anticipation,” “disgust,” “fear,” “joy,” “sadness,” “surprise,” “trust,” “negative,” and “positive.” The results by fitting all the count models are shown in Table 1 with outcomes “retweet counts,” and “favorite (like) counts.” The details of estimates and *p*-values are listed in Table 2.

**Table 1:** Variable selection in models of retweet and favorite counts with sentiment scores.

**Models**	**Poisson**	**Negative binomial**	**Zero-inflated (binomial logit link)**	**Comments**
Variables found significant for retweet counts	“anger,” “anticipation,” “disgust,” “fear,” “joy,” “sadness,” “surprise,” “trust,” “negative,” “positive”	“disgust,” “joy,” “sadness,” “surprise,” “trust,” “negative,” “positive”	“anticipation,” “joy,” “sadness,” “trust,” “positive”	Negative binomial model seems to be the best performing model.
AIC	401600	33291	N/A	
Variables found significant for favorite counts	“anger,” “anticipation,” “disgust,” “fear,” “joy,” “sadness,” “surprise,” “trust,” “negative,” “positive”	“anger,” “anticipation,” “disgust,” “fear,” “joy,” “sadness,” “surprise,” “trust,” “negative,” “positive”	“positive”	Negative binomial model seems to be the best performing model.
AIC	1462000	57555	N/A	

N/A, Not applicable.

**Table 2:** Estimates (confidence intervals) from negative binomial regression models.

	**Outcome** = **Retweet counts**				**Outcome** = **Favorite counts**			
**Variables**	**Estimate**	**2.50%**	**97.50%**	***P*-value**	**Estimate**	**2.50%**	**97.50%**	***P*-value**
(Intercept)	5.189095	4.658966	5.793146	1.51E-218	20.61177	19.03433	22.35081	0
Anger	1.102365	0.970846	1.253846	0.141028043	1.162999	1.053729	1.284773	0.002478
Anticipation	1.065537	0.985909	1.153526	0.149869222	1.102797	1.039483	1.171189	0.003221
Disgust	0.728357	0.632539	0.840187	4.04E-06	0.838794	0.754632	0.933522	0.000688
Fear	0.921362	0.838073	1.013359	0.109780306	0.916722	0.854627	0.983612	0.024308
Joy	0.81454	0.709756	0.935488	0.002939532	0.886361	0.801846	0.980654	0.020245
Sadness	1.212522	1.093228	1.345493	0.00056631	1.215866	1.125497	1.313882	3.53E-06
Surprise	0.709527	0.614592	0.82223	4.02E-06	0.783444	0.702947	0.874976	1.30E-05
Trust	1.109723	1.007908	1.223671	0.027799061	1.086922	1.01049	1.170196	0.019462
Negative	1.181363	1.086161	1.287272	0.00017715	1.134959	1.068147	1.207219	0.000158
Positive	1.140512	1.054933	1.234997	0.000229091	1.093359	1.032497	1.158825	0.000907

## Discussion

The study aims to describe the prevalence of long-haul COVID-19 using among the local (US Twitter data regions) and extrapolate our estimates to the global population. For future work, continuous variables can be presented by summary statistics (i.e., mean, median, standard error, range, 95% CI, and correlations) and the categorical variables by frequency distributions (i.e., frequency counts, percentages, and 95% CI). Simple linear regression, logistic regression, and Cox univariable and multi-variable regression analyses can be performed to answer data-specific questions. Survival curves for overall survival probability can be generated using Kaplan–Meier curves. Bayesian variable selection and machine learning algorithms can detect the causes related to the outcome of interest. The chi-square test can be used to understand the association between two factors. The results can be declared significant at a significance level of 5% in the hypothesis testing sense and also with credible Bayesian intervals. The focus should be on building an appropriate prediction model that can incorporate various features, such as vital signs, demographics, and genetic factors, to predict the possibility of occurring long COVID-19 occurrence, including COVID-19-related death prediction during the early stage of the study. A major initiative has been undertaken with the L3C data challenge (Announcing The NIH Long COVID Computational Challenge, 2022). The models developed may have both frequentist and Bayesian counterparts to address the uncertainties in the prediction algorithms. The model needs to have inherent flexibility so that it can be modified according to the targeted population and outcomes, thus identifying the best set of predictors.

We can also address the progression of the disease through survival models. The solution to this problem involves determining the stages of disease progression and predicting the outcomes. The International Council for Harmonization Efficacy guideline (ICH E9) discusses subgroups and recommends identifying potential subgroups prior to the analysis to investigate treatment heterogeneity. So by identifying the factors involved in predicting the long-term outcome of COVID-19, we will be able to identify the subgroups of patients who are at risk of such outcomes. Then, targeted treatment and medical resource allocation can be implemented for patients in different stages of the disease. Furthermore, we can identify the subgroup of patients who have similar outcomes, demographics, lifestyle, or risk factors. Our results are general enough so should be applicable to a majority of countries. ClinicalTrials.gov is a repository of registered clinical trials across the globe. We do acknowledge that there are other registries specific to countries, including European Union, but ClinicalTrials.gov does provide a good representation of planned clinical/observational studies from across the world.

Twitter is a global social network and so represents emotions/sentiments across the globe. Since our Twitter sample size is reasonably large, the results should be applicable to different parts of the world. We did not attempt to analyze Twitter data from different languages. We note that a recent study by Lee and Colautti (2022) did utilize non-Arabic words to assess the effect of ISIS in creating confusion and panic during COVID-19. Twitter does support 34 languages; investigating emotions/sentiments in any of the 34 languages will be a project in itself.

We intend to provide code to future researchers to extract information from Twitter so that this could help them if they want to design studies relating to long COVID. The researchers could apply the code and extract information on an ongoing basis from Twitter which would be helpful in designing studies. Also, researchers can follow our methodology to extract information from ClinicalTrials.gov. In fact, the basis of our research framework is to provide a basic foundation for designing long COVID studies in a “seemingly adaptive” way.

This research discusses reshaping several limitations and research ideas on a single platform.

EHR and clinical trial data, on both adult and pediatric populations, are essential to address the following research questions. The research questions that can be addressed are follows:

What is the prevalence of the long-term effects of COVID-19 on patients who have recovered from the disease? What are the demographic and other characteristics of long-haul COVID-19 patients?

What factors other than the COVID-19 infection including demographics (sex, age, race), vaccination status, concomitant therapy, hospitalization, life support or intensive care unit, and comorbidities that could influence long-term COVID-19? Are any genetic, gene-treatment, or gene-environment factors that work as effect modifiers responsible for long COVID?

Brain fog is one of the long-term symptoms, and there are growing concerns about neurological disease progressions as a long-term consequence of COVID-19. The effect of such a claim can be verified by health data through this research. Were patients recovering from intensive care or life support due to hospitalization faster compared to patients with or having comorbidities?

Identify the subgroups of adult and pediatric patients that are more prone to the long-term effects of COVID-19 and mortality. How anti-virals and current medications be utilized for potential benefit to such long haulers of COVID-19?

The work can help to address several unaddressed research questions, thus, in turn, will help implement such prediction methods to address issues of designs in live clinical trials. The novel techniques and methodologies will help improve efficiency and identify subgroups that could benefit from personalized treatment. Thus, in turn, the hands-on software can be utilized by clinicians and researchers to recommend doses, sample size, and power and address the shortcomings in current trials, thus contributing to the potential approval of therapies and vaccines for long-term efficacy and prevention. Each design has its pros and cons and assumptions that must be justified and verified. Similarly, who is at risk of long COVID and what the future entails for long COVID patients is important to understand. We possibly will deal with a sicker population in the coming years, and we need healthcare resources to combat the post-pandemic lingering post-acute sequelae of COVID and other healthcare needs. The study of long COVID or broadly COVID survivorship is one of the areas where biostatisticians have a great deal to contribute in the coming years (Mukherjee, 2022), targeting a population of more than 100 million affected worldwide, with 44 million people in the United States. According to the current CDC, the current estimates show that 13.3% of the people with COVID-19 have lasting effects at 1 month or longer after infection 2.5% at 3 months or longer, based on self-reporting, and more than 30% at 6 months among patients who were hospitalized. This shows that there is a potential market for drug development in this population with unmet research needs. The health effects of COVID-19 appear to be prolonged and can exert marked stress on the healthcare system. To understand the healthcare needs of the long-haul COVID-19 vulnerable population, research needs, dedicated clinics, and segregation of hospitalization facility needs are imperative to help the millions recovering from this disease. This multidisciplinary research article will encourage conducting clinical trials and channelizing the efforts toward long-term COVID-19-related therapeutic interventions to mitigate the adverse physical and mental health effects among the patients (Yelin et al., 2020).

## Conclusion

Long COVID-19 can potentially produce a second public health crisis. It is imperative to take sufficient measures to curb this condition and assess its severity before it escalates and spreads to a higher level. The global health community needs to be aware of the importance of research in long COVID-19.

## Data availability statement

Publicly available datasets were analyzed in this study. This data can be found at: https://clinicaltrials.gov.

## Author contributions

AB has contributed to the concepts, methods, and authoring of the paper. AS and SR have contributed to the overall writing manuscript through valuable comments and concepts. All authors contributed to the article and approved the submitted version.
